# The Gut Microbiome Role in Multiple Myeloma: Emerging Insights and Therapeutic Opportunities

**DOI:** 10.3390/hematolrep17060056

**Published:** 2025-10-27

**Authors:** Mina Y. George, Nada K. Gamal, Daniel E. Mansour, Ademola C. Famurewa, Debalina Bose, Peter A. Messiha, Claudio Cerchione

**Affiliations:** 1 Department of Pharmacology and Toxicology, Faculty of Pharmacy, Ain Shams University, Cairo 11566, Egypt; 2 Medical School, University of Minnesota, Minneapolis, MN 55455, USA; 3 Department of Medical Biochemistry, Faculty of Basic Medical Sciences, College of Medical Sciences, Alex Ekwueme Federal University, Ndufu-Alike Ikwo, Abakaliki P.O. Box 1010, Ebonyi, Nigeria; 4 Advanced Technology Development Centre, Indian Institute of Technology, Kharagpur 721302, India; 5 Department of Internal Medicine, Rutgers New Jersey Medical School, Newark Medical Center, RWJBarnabas Health, Newark, NJ 07101, USA; 6 Hematology Unit, Istituto Romagnolo per lo Studio dei Tumori “Dino Amadori”—IRST IRCCS, Via Piero Maroncelli 40, 47014 Meldola, FC, Italy

**Keywords:** gut microbiome, multiple myeloma, inflammation, systemic immunity, metabolites, dysbiosis

## Abstract

Multiple myeloma is a hematological cancer depicted by the proliferation of plasma cells within the bone marrow, causing immune dysfunction and other abnormalities. The gut microbiome, the microbial community in the gastrointestinal tract, was found to modulate systemic immunity, inflammation, and metabolism. Although the interplay between gut microbiome and multiple myeloma has been found in recent research, there is a gap in knowledge linking the effect of the microbiome on the pathogenesis and treatment of multiple myeloma. The imbalance in the gut microbiome, dysbiosis, may influence multiple myeloma pathogenesis through immune modulation and inflammation. Certain microbial species have been associated with multiple myeloma progression, complications, and therapeutic responses to treatment. Moreover, microbiome-derived metabolites, short-chain fatty acids, can influence the immune circuits associated with multiple myeloma progression. Understanding the bidirectional relationship between multiple myeloma and gut microbiota may provide insights into enhanced treatment and the development of new microbiome-based interventions. The current review provides a comprehensive highlight of current evidence linking the gut microbiome with multiple myeloma, demonstrating its significant roles in the development, progression, and treatment of multiple myeloma. Additionally, it focuses on the therapeutic potential of modulating the gut microbiome as a novel adjunct strategy in multiple myeloma management.

## 1. Introduction

### 1.1. Multiple Myeloma

Multiple myeloma (MM) is a hematologic neoplastic disease characterized by clonal proliferation of plasma cells in the bone marrow [1]. It is the second most common hematological cancer of the plasma B cells, with significant heterogeneity in the bone marrow microenvironment, and the most common primary bone malignancy [2,3]. According to the International Myeloma Working Group (IMWG), MM is defined as a clonal plasma cell malignancy characterized by clonal bone marrow plasma cells ≥ 10% or extramedullary plasmacytoma and evidence of end-organ damage attributable to the plasma cell disorder (the classic CRAB features), including hypercalcemia (serum calcium > 0.25 mmol/L above the upper limit of normal), renal insufficiency (serum creatinine > 2 mg/dL or creatinine clearance < 40 mL/min), anemia (hemoglobin value of >20 g/L below the lowest limit of normal, or a hemoglobin value < 100 g/L), and bone lesions (one or more osteolytic lesions) [4,5].

It exerts severe sequelae of consequences, including severe bone pain, bone loss, and pathological fractures that considerably reduce patients’ quality of life and affect their prognosis. This bone dysfunction includes persistent inhibition of osteoblastic bone formation and excessive osteoclast resorption that result in incurable osteolytic lesions even when patients are in complete and prolonged remission [2,6].

MM has been studied as part of a disease of plasma cell disorders. The earliest stage is monoclonal gammopathy of undetermined significance (MGUS), diagnosed by serum monoclonal protein < 3 g/dL, <10% clonal bone marrow plasma cells, and absence of end-organ damage, with an average risk of progression to MM of ~1% per year. Smoldering multiple myeloma (SMM) represents the intermediate stage. SMM is characterized by serum M-protein ≥ 3 g/dL and/or 10–60% clonal plasma cells in the bone marrow, without CRAB features, but with a higher risk of progression to MM (~10% per year). Symptomatic or active MM is defined by ≥10% bone marrow plasma cells or extramedullary plasmacytoma, in addition to CRAB features. Plasma cell leukemia and extramedullary plasmacytoma represent the most aggressive progression of MM relapsed/refractory states [7].

The etiology of MM remains largely unclear. However, the pathophysiology of MM is complicated, with high heterogeneity underpinned by a multistep process of initiation and progression implicated to be triggered by environmental and genetic aberrations. Nevertheless, MM may develop without known risk factors and be promoted by multiple factors. The initiation of the asymptomatic premalignant stage is recognized as MGUS. In MGUS, the clonal plasma cells produce light chain immunoglobulin (Ig) and the absence of monoclonal heavy chain Ig expression. Light chain MGUS, a precursor to clinical MM, is thus characterized by an abnormal κ/λ FLC ratio, and patients with MGUS IgG or IgA progress to MM [8,9]. A more advanced asymptomatic stage is SMM, which may progress to active MM within 5 years. It is shrouded in genetic alterations, mutation, chromosomal translocation, aneuploidy and epigenetic aberrations [10]. The levels and patterns of these genetic and epigenetic changes are related to patients’ survival, the progression time from MGUS through SMM to symptomatic MM, the time between initiation and progression, and response to standard treatment. Globally, variation in the levels of DNA methylation is common among patients, and progression to MM is associated with hypomethylation when compared to premalignant stages [4]. However, the perturbation of intricate signaling pathways follows the genetic aberrations. Some of these pivotal signaling pathways include the mitogen-activated protein kinase (MAPK) pathway, nuclear factor kappa B (NF-κB) pathway, and cell cycle pathway, contributing to the development of MM [11]. NF-κB is considered the key player of inflammation in human bodies that represents the connection between different signaling axes [12,13]. The role of the NF-κB pathway and its regulator kinase and receptor CD40 in lymphoid neoplasms and MM is well reported in the existing literature [14]. In addition, the stroma cells in the bone marrow microenvironment (BMM) contribute to the pathogenesis of MM. Bone marrow stroma cells (BMSCs) orchestrate binding to a number of proteins expressed on MM cells to enhance their retention in the BMM. Further interactions of MM cells with BMM induce secretion of soluble growth factors and cytokines, including insulin-like growth factor (IGF-1), vascular endothelial growth factor (VEGF), B-cell activating factor (BAFF), a proliferation-inducing ligand (APRIL), interleukin-6 (IL-6), and tumor necrosis factor-α (TNF- α) [11]. The integrated secretion of these proteins in the BMM activates intracellular signals that regulate the growth, proliferation, migration, and drug resistance of malignant MM cells [8,15,16].

These malignant bone marrow plasma cells secrete excessive amounts of monoclonal immunoglobulin paraproteins, leading to hematopoietic dysfunction, bone deterioration, and end-organ failure [8]. Of importance, the preponderance of monoclonal immunoglobulin (paraprotein or M-protein) secreted by malignant plasma cells causes hyperviscosity, amyloidosis, fatigue, and recurrent infections. The monoclonal immunoglobulin infiltrates vital organs, causing renal dysfunction [8].

The global incidence of MM has steadily increased by 143% since 1975, making it the second most prevalent blood cancer following leukemia, with the attendant high morbidity and mortality [8,17]. Global deaths due to neoplasms increased by 94% from 1990 to 2016. According to the Global Cancer Observatory (GLOBOCAN) statistics, there were 160,000 cases of MM globally in 2018, accounting for 0.9% of all cancer diagnoses, and about 90,000 of those cases were male and 70,000 were female [18]. In the latest GLOBOCAN estimates, there were 176,404 new cases of MM globally, with 117,077 new deaths from MM in 2020 [19]. These estimates represent 0.9% newly diagnosed cases and 1.2% new deaths of all reported cancer types globally. MM contributes to up to 10% of hematologic neoplasms. Less than two-thirds of people under 40 seem to experience it more regularly than people over 40, and the median age of diagnosis is 65 years [8].

Currently, MM is an insidious and intractable disease; relapses and drug resistance still remain significant challenges and an undaunting source of worry to clinicians. However, recent advancements in understanding the molecular and cellular background of MM have led to the development of effective chemotherapy, stem cell transplantation, proteasome inhibitors (PI), immunomodulatory drugs (IMiDs), monoclonal antibodies, bispecific antibodies, and chimeric antigen receptor T-cell immunotherapy (CAR-T) [18,20]. The main goals of treatment of MM are to block disease progression, suppress malignancy and metastasis, mitigate MM-related complications, and increase survival. The approval of multiple active agents in the treatment of MM has resulted in a number of drug combination regimens that can be used first-line and in relapsed settings. A triplet combination regimen may be considered for MM patients ineligible for stem cell transplantation and then followed with maintenance therapy until toxicity limits the use. The foremost frontline triplet therapy is RVd (lenalidomide, bortezomib, and dexamethasone), which is the current standard for initial treatment [21,22]. Other proteasome inhibitors can be used for MM treatment, including carfilzomib [23]. However, carfilzomib treatment has been associated with several adverse effects [24]. The new generation proteasome inhibitors and IMiDs, including ixazomib, pomalidomide, isatuximab-containing regimens, and chimeric antigen receptor (CAR) T-cell therapy, are being used to manage relapses with evident efficacy [10,25]. Despite significant improvements in the clinical treatment strategies and increased survival rates of MM patients, MM remains incurable due to inevitable relapses and drug resistance during maintenance therapy.

### 1.2. The Gut Microbiome

Human microbiota is the ecological community of commensal, symbiotic, and pathogenic microorganisms colonizing the human body, including the GIT, respiratory system, oral cavity, skin, and female reproductive system [2,26]. It offers many health benefits to the host, including immunity regulation and response, reaction to infection, energy metabolism and nutrient metabolite uptake, hematopoiesis and neurobehavioral traits, GIT maintenance and integrity, and biosynthesis of steroids, vitamins, neurotransmitters, and hormones [20,27,28,29]. The human gut is a natural habitat that harbors billions of microorganisms, which form a complex ecosystem known as the gut microbiota [27]. Although the composition of microbiota is compositionally dynamic throughout life, infancy and early childhood shape the microbial population, which is still vulnerable to changes by diseases and antibiotics. Bacteria in the phyla of *Actinobacteria*, *Bacteroidetes*, *Firmicutes*, and *Proteobacteria* are the most studied microorganisms in the gut microbiota [30]. The genomic content (microbiome) of microbiota is about 100 times the human genome [28,31]. However, growing evidence indicates the role of human microbiota in the development of cancers, infections, inflammatory, and immune-mediated disorders [28,31]. The gut microbiota was associated with cancer for the first time in 1970 when germ-free mice subject to 1,2-dimethylhydrazine had less tumor load [32]. Later studies show that tumorigenesis is ameliorated in germ-free or antibiotic-treated mice [30,33,34]. Studies have shown that the microbiota can affect immune responses related to the innate and adaptive immune system via microbial signals, immune cells, cytokines, chemokines and T-cell homeostasis [35,36]. The pathogenesis of hematological neoplasms, including lymphoma, acute leukemia, and MM, has been associated with human microbiota. The increasing research effort to understand the pathophysiological machinery underlying the development of MM implicates the gut microbiome [20]. The emerging mechanistic and signaling cascades for this crucial role implicate gut microbiome taxonomic shift, influence of gut microbiome on the host’s adaptive and innate immune systems, inflammatory pathways and bone marrow microenvironment [37,38]. Gut microbiome dysbiosis, that is, imbalanced composition and diversity of the gut microbiome, may provoke activation of plasma cells in the bone marrow, leading to clonal selection, genetic translocation and oncogenesis. Microbiome-synthesized bioactive compounds, such as short-chain fatty acids, are capable of modulating NF-κB, inflammatory cytokines and T-helper cells to affect MM pathogenesis [37]. Activation of T-helper 17 (Th17) cells in the gut may migrate to bone marrow and promote myeloma progression [39]. The gut microbiome affects the differentiation and migration of Th17 cells in the bone marrow. Alterations in short-chain fatty acids occasioned by altered gut microbiota composition are associated with MM progression [40]. The findings that the gut microbiome may promote inflammation and/or stimulate the release of immunosuppressive interleukins indicate its pivotal role in the development and progression of MM and therapeutic options, including transplantation [17]. The development of gut microbiota-targeted immunotherapy, surfacing in the innovative approach to treating MM, underscores the immunological connections between the gut and the transition from asymptomatic MGUS to symptomatic MM.

Therefore, this review is targeted at uncovering the role of the gut microbiome in the pathophysiology of MM, immunological responses, potential mechanisms of gut microbiome-mediated perturbations to the progression of MM and the predominance of certain bacterial taxa in the gut microbiome implicated in driving MM carcinogenesis in recent studies. In addition, we present emerging novel therapeutic strategies, including probiotics, prebiotics, and personalized medicine.

## 2. Methods

The literature added in the current review was identified through searches in PubMed and Scopus. Articles published between 2020 and 2025 were considered, with emphasis on experimental and clinical studies. Additional references were identified from the bibliographies of relevant papers. The search terms used were Multiple myeloma, Microbiome, Microbiota, Clinical, and Preclinical. We focused our search nearly on the last 5 years. The number of articles screened may be around 40 to 50 articles.

## 3. Microbiome and Hematologic Cancers

This section discusses how gut microbiome alterations can contribute to the pathogenesis and treatment outcomes of hematologic cancers, including multiple myeloma. Also, how gut microbiota can affect systemic immunity and inflammation. This could affect the disease progression and treatment of hematologic malignancies.

### 3.1. Role of the Microbiome in Hematological Cancers

The gut microbiome plays a crucial role in pathogenesis and the therapeutic response of hematologic malignancies. Alteration to microbial diversity, or dysbiosis, has been correlated with disease progression and poor clinical outcomes. That is, microbial signatures can influence not only the incidence of hematologic cancers but also treatment tolerance and relapses. For instance, the gut microbiome has been proven as a biomarker for clinical outcomes in allogeneic hematopoietic stem cell transplantation and its manipulation has also been shown to boost therapeutic responses in animal models [41]. The role of the microbiome composition has also been highlighted in a study that aimed to explore the effects of alterations in the gut microbiome on infections and immunological sequelae during treatment of Korean children, adolescents and young adults with hematologic malignancies. 16S rRNA gene sequencing of 26 oncohematological patients showed a lower abundance of *Lachnospiraceae* and a higher abundance of *Enterococcaceae* in their gut microbiome than their healthy counterparts, demonstrating a relationship between microbial configuration and hematologic cancers [42].

### 3.2. Systemic Immunity and Inflammation

The gut microbiome has a well-established impact on systemic immunity and inflammatory processes. Alterations in the gut microbial composition have the capacity to stimulate inflammatory signaling pathways that extend beyond the scope of the gut, impacting systemic immunity as a whole. That is, dysbiosis of the microbiome has been associated with multiple inflammatory and autoimmune diseases such as IBD, multiple sclerosis, type I diabetes, and rheumatoid arthritis, suggesting a role of the microbiome in inducing inflammatory changes on a systemic level [43].

In addition to pro-inflammatory effects, alterations in the gut microbiome interfere with the immune system function, susceptibility to infection, and vaccine response impairment. Studies utilizing germ-free (GF) mice showed an impaired immune system in GF mice compared to their gnotobiotic counterparts. Specifically, GF mice lacked proper maturation of the gut-associated lymphoid tissues and isolated lymphoid follicles and developed relatively fewer and smaller Peyer’s patches and mesenteric lymph nodes. Additionally, the intestines of GF mice had immature and defective IgA-producing B cells, intestinal epithelial cells with a lower regenerative rate, and a thinner protective intestinal mucosal layer than gnotobiotic mice, which subjected them to a higher susceptibility to pathogen infections. On a molecular level, GF mice had decreased levels of interleukin-17-producing Th17 cells and defects in regulatory T cells. Of note, some of the immunologic developmental impairment, namely the defective IgA-producing B cells and improperly developed lymphoid structures were, however, reversed with the sustained exposure of GF mice to commensal bacteria, further reinforcing the role of the microbiome in the proper development of the immune system and suggesting a dynamic relationship between them [35,44].

The gut microbiome activated the innate immune system. It constantly triggers the release of IL-10 from resident macrophages, leading to the stimulation of T cells and the inhibition of Th17 cells, which maintains the function of the intestinal innate immune system. An alternative mechanism through which intestinal commensals regulate innate immunity is through boosting the bone marrow neutrophil response against certain pathogens, such as *Streptococcus pneumoniae* and *Staphylococcus aureus* [45].

### 3.3. The Effect of Gut Microbiome on the Immune System

There appears to be a myriad of mechanisms through which the microbiome influences the immune system, potentially leading to carcinogenesis. While the formation of a local chronic inflammatory state is regarded as the most implicated carcinogenic mechanism, some bacteria, such as *Helicobacter pylori*, induce DNA damage and disrupt essential intracellular signaling pathways involved in regulating mucosal cell growth and proliferation. Helmink et al. further explored the different mechanisms underlying dysbiosis-mediated colorectal cancer, ranging from the production of pro-inflammatory toxins and the induction of reactive oxygen species to interference with signaling pathways and the impairment of antitumor immune functions.

*Bacteroides fragilis* has been shown to induce colitis in mice, interfere with E-cadherin junctions, and promote β-catenin signaling and IL-8 release in epithelial cells of the colon through its metalloprotease *B. fragilis* toxin [46]. *Fusobacterium nucleatum* has been shown to promote tumorigenesis by aiding tumors in evading the antitumor innate immune system due to the interaction between the *Fusobacterium nucleatum* Fap2 protein and the NK cell inhibitory receptor TIGIT [47]. Furthermore, *Fusobacterium nucleatum* was shown to induce small intestinal and colonic carcinogenesis, increase infiltration of myeloid cell subsets into tumors and activate NF-kB pro-inflammatory signaling in genetically predisposed mice [48]. Apart from immune dysregulation and alterations in signaling pathways, *Escherichia coli* and *Campylobacter jejuni* have proven to promote carcinogenesis through the production of directly genotoxic metabolites colibactin and cytolethal distending toxin, respectively.

**Figure 1:**
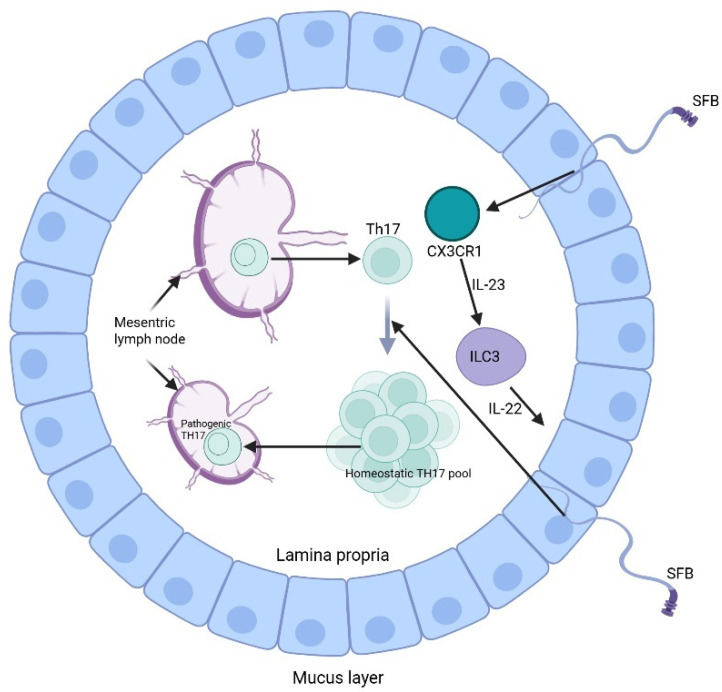
Induction of Th17 by segmented filamentous bacteria (SFB) (Created in https://BioRender.com).

Other molecular mechanisms have been elucidated by Honda et al., showing a relationship between the microbiome and the adaptive immune system, namely Th17 and regulatory T-cells. Specifically, the epithelium-adhering microbial species promotes Th17 differentiation and polarization through binding to the intestinal epithelial cells and presenting its antigens on APCs to naïve CD^4+^ T cells. Differentiated Th17 cells can then release cytokines such as IL-17, which plays a role in epithelial barrier integrity against pathogens. However, certain microbes like segmented filamentous bacteria can trigger Th17-mediated autoimmune responses by inducing epithelial expression of serum amyloid A. Serum amyloid A promotes the release of IL-23 from CX3CR1-expressing cells that can lead to the conversion of differentiated TH17 into pathogenic TH17 that can migrate to the draining lymph nodes, inducing autoimmune disease (Figure 1) [49,50].

Furthermore, the gut microbiome plays a crucial role in establishing immune tolerance through its ability to promote regulatory T cell development in the intestinal lamina propria. The colon contains a specific subset of Treg cells, which express the RORγt transcription factor and need microbiota signals for their development [51]. The absence of microbiota in germ-free mice results in a substantial decrease in peripherally induced Treg cells, which demonstrates the essential role of microbial communities in their development. The RORγt-expressing Treg cells show restricted T cell receptor (TCR) diversity and develop from specific microbial antigens instead of thymic selection processes. Research on TCRs from colonic Treg cells shows that their development and maturation process happens within the colon and needs microbial stimulation. The research demonstrates how the microbiome creates an immune system that adapts to maintain Treg cell populations essential for intestinal health and inflammation prevention [49].

### 3.4. Link Between the Gut Microbiota and Other Cancers

The relationship between microbiome and carcinogenesis has been witnessed in a broad range of cancers. For instance, *H. pylori* has been implicated in both gastric adenocarcinoma and mucosa-associated lymphoid tissue (MALT lymphoma), which earned it a classification by the World Health Organization as a class I carcinogen [43]. Colon carcinogenesis has also been found to be heavily influenced by different species of the microbiome. A study conducted by He et al., for example, highlighted the role of *Campylobacter jejuni* in the formation of colorectal cancer by altering the microbial composition through the DNAse activity of the genotoxin cytolethal distending toxin [52]. A large body of evidence has also reported an association between the aforementioned *Fusobacterium nucleatum* and both colorectal carcinoma and adenoma [48,53,54]. In a study conducted by McCoy et al., a higher abundance of *Fusobacterium* in the normal rectal mucosa was significantly associated with a higher likelihood of the presence of adenomas, and greater local cytokine gene expression, hinting at a potential role of mucosal inflammation underlying the process [55].

Other cancers that were proven to be associated with alterations in the microbiome include hepatocellular carcinoma (HCC), biliary tract, and pancreatic cancers [56]. *Helicobacter hepaticus* was shown to induce the development of HCC through promoting tumor growth and stimulating the WNT and NF-kB signaling pathways in mice [57]. Helicobacter species has also been associated with HCC in clinical studies that were able to find Helicobacter in the liver of patients with primary liver carcinoma and not in their healthy counterparts [58]. The fecal abundance of another microbial species—*Escherichia coli*—was also found to be higher in the presence of HCC, hinting at a potential link between *Escherichia coli* and carcinogenesis of the liver and further supporting the role of the microbiome in carcinogenesis in general [59].

Gallbladder carcinoma is another cancer where an alteration in the microbiome has been demonstrated to play a role in its pathogenesis. *Salmonella typhi* infection has been proven as a causative agent of gallbladder carcinoma, as it induces tumorigenesis of predisposed cells with TP53 mutations and c-MYC amplification through stimulating MAPK and AKT pathways [60]. Higher risk of biliary tract cancers has been associated with multiple alterations in different microbial species, such as higher abundance of *Helicobacter* species, *Methylophilaceae*, *Fusobacterium*, *Prevotella*, *Actinomyces*, and *Novosphingobium* [56].

The effect of microbiomes on cancer development extends to include pancreatic cancer. A comprehensive comparison between the salivary microbiota of patients with pancreatic cancer and healthy controls demonstrated a higher prevalence of *Neisseria elongata* and *Streptococcus mitis* in the saliva in the pancreatic cancer cohort [61]. Similarly, a case–control study showed that a higher abundance of salivary *Porphyromonas gingivalis* and *Aggregatibacter actinomycetemcomitans* incurs the same association [62].

## 4. Interplay Between the Gut Microbiome and Multiple Myeloma

Recently, attention has shifted to the microbiota’s potential role in MM. Research shows that symbiotic microorganisms have impacts beyond the gastrointestinal tract, not only modifying local immune responses through metabolites and interacting with innate immunity, but also affecting systemic immunity by affecting the function and differentiation of immune cells, suggesting the role of the microbiome in regulating the immunological environment linked to the MM pathophysiology [63]. The main microbial groups associated with MM have been reported in Table 1.

### 4.1. Microbiota Alteration in Multiple Myeloma

Investigation on mouse models showed that the microbiota may contribute to the development of MM. In humans, four bacterial phyla are the most abundant, which are *Firmicutes*, *Bacteroidetes*, *Actinobacteria*, and *Proteobacteria* [67]. The microbiota of MM patients differs from that of healthy people, which is known as microbiota dysbiosis [38,64]. Patients with MM have demonstrated an increased abundance of *Proteobacteria* and a decrease in *Actinobacteria*. Moreover, stool samples of MM subjects showed increased levels of *Streptococcus*, *Klebsiella*, and *Pseudomonas aeruginosa* [20]. *Anaerostipes hadrus*, *Clostridium butyricum*, and *Clostridium saccharobutylicum* were found to be substantially less prevalent in MM patients than in healthy individuals [64,68,69]. Additionally, adding *Clostridium butyricum* to an MM mouse model resulted in tumor progression remission. These alterations in microbial composition may affect the microbiota-derived metabolites, which are important in signaling and are considered metabolic substrates. Such alterations can disrupt immune regulation and metabolic processes, hence resulting in disease progression [20].

### 4.2. Microbiota-Derived Metabolites in Multiple Myeloma

#### 4.2.1. Short-Chain Fatty Acids

The metabolites generated by the gut microbiota from the fermentation of insoluble dietary fibers are the short-chain fatty acids (SCFAs), mainly consisting of acetate, propionate, and butyrate [68]. They are important for intestinal homeostasis and immune regulation via influencing signaling pathways [69]. SCFAs can activate NF-kB and produce pro-inflammatory cytokines, which include IL-6 and tumor necrosis factor alpha (TNF-α), which contribute to Th17 cell development. Moreover, SCFAs also increase IL-10 levels and forkhead box P3 (FoxP3) expression, causing CD^4+^ T cell differentiation to Treg cells [70]. Butyrate, which has been known for its anti-inflammatory effect, can hinder NF-κB activation by lipopolysaccharide (LPS) and also inhibit neutrophils induced by LPS from releasing pro-inflammatory cytokines, such as cytokine-induced neutrophil chemoattractant-2, TNF-α, and nitric oxide [71]. After myeloma treatment, researchers discovered that negative microscopic residual lesions were linked to butyrate, generated by *Eubacterium halii* or *Faecalibacterium prausnitzii* [66].

#### 4.2.2. L-Glutamine

A metabolite that is also linked to gut microbiota is L-glutamine. Glutamine can enhance intracellular energy production and also fulfill the increased demand for nucleotide biosynthesis in cancer cells to promote growth, since it is utilized as a nitrogen or carbon source [72,73]. The metabolism of MM cells consumes an excess amount of glutamine, and so their growth is hampered by the depletion of glutamine [74,75]. It was found that stool samples from MM subjects had considerably higher levels of bacteria associated with nitrogen utilization and recycling, such as *Klebsiella* and *Streptococcus*, compared to healthy individuals. After transplanting *Klebsiella pneumoniae* into a mouse model of MM, researchers found that bacteria contribute to disease progression through glutamine de novo synthesis. The investigation demonstrated that mice treated with ammonium or urea had substantially higher levels of glutamine concentrations in their serum and feces. The accumulation of abnormal amino acids and blood urea in the host bone marrow microenvironment supported the proliferation of nitrogen-cycling microbiota, leading to the degradation of urea and glutamine synthesis [64]. These findings suggest that gut microbiota may offer multiple myeloma cells a supply of nitrogen to fulfill their energy needs for rapid proliferation and glutamine production, which would boost the progression of the disease.

### 4.3. Microbiota and Immune Regulation in Multiple Myeloma

Intestinal dendritic cells detect and present antigens from colonized bacteria in immune tissues such as the Peyer’s patches and mesenteric lymph nodes. Moreover, this cell also differentiates T cells in lymph nodes into Treg, Th17, Th1, and Th2 cells, producing pro- or anti-inflammatory cytokines [76,77]. The interaction between the gut microbiome and the immune system affects both immune regulation and tumor growth. Excessive Th17 cell inflammation or Treg-induced immune suppression can promote cancer progression [78]. The intestinal Th17 cell differentiation aids in protecting the mucosal barrier from pathogens. In turn, commensal bacteria in the gut assist in Th17 cell differentiation [79]. These cells are distinguished by the generation of cytokines, including IL-17A, IL-17F, and IL-22, which are inflammatory mediators. Among them, IL-17 can accelerate multiple myeloma progression by promoting the growth of tumors by influencing the tumor microenvironment [80,81].

**Figure 2:**
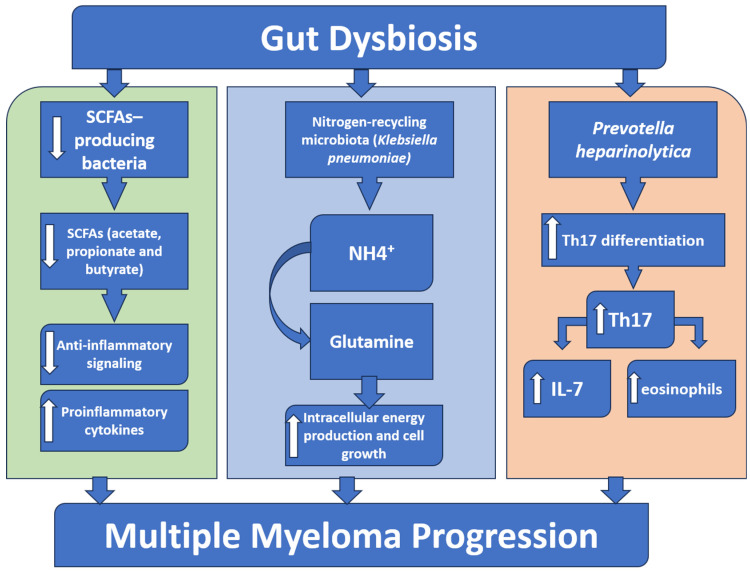
A schematic diagram showing the role of gut microbiota in multiple myeloma progression. Gut dysbiosis results in a reduced abundance of the beneficial short-chain fatty acids (SCFAs)-producing bacteria, which leads to a decrease in the anti-inflammatory SCFAs, permitting an overactivation of inflammatory signaling. Simultaneously, an increase in nitrogen-recycling bacteria (e.g., *Klebsiella pneumoniae*) increases glutamine synthesis that boosts the intracellular energy and tumor growth. Furthermore, a specific bacterium like *Prevotella heparinolytica* supports the intestinal Th17 cell differentiation and migration to the bone marrow, where they generate IL-17, stimulate eosinophils, and trigger additional IL-6 release. Together, these pathways all lead to the progression of multiple myeloma.

Interestingly, a preclinical investigation has shown that the gut commensal bacterium Prevotella heparinolytica stimulates Th17 cell differentiation in the intestine, which may then migrate to the bone marrow, contributing to multiple myeloma progression. In genetically engineered Vk*MYC mice, disease progression was substantially delayed when either the gut microbiota was disrupted or IL-17 was deficient [39,82]. This study suggested that there is an immunological axis between IL-17 and eosinophils. Mechanistically, it was discovered that IL-17 in plasma cells stimulates signal transducer and activator of transcription 3 (STAT3) signaling and activates eosinophils, thus boosting the pro-tumor environment. It was found that administering antibodies that target IL-17, its receptor IL-17RA, and IL-5 can disrupt this axis and significantly decrease the number of Th17 cells and eosinophils, hence preventing the progression of multiple myeloma and indicating a promising therapeutic approach (Figure 2) [39].

## 5. Role of the Microbiome in Multiple Myeloma Treatment

Owing to their dual mechanisms, IMiDs (i.e., lenalidomide and pomalidomide) are highly considered for MM treatment [83]. Their multifaceted mechanisms of action involve direct tumoricidal effects, modulation of the bone marrow microenvironment, and enhancement of host immune surveillance. IMiDs have the ability to enhance the immune system to attack cancer cells by binding to the E3 ubiquitin ligase complex, causing the breakdown of the transcription factors important for myeloma plasma cell survival. Inhibition of the survival of myeloma cells can lead to apoptosis, inhibition of pro-myeloma cytokines such as IL-6, and the prevention of cancer progression. In addition, IMiDs promote IL-2 release and T cell activation, inducing significant immunostimulatory effects [84]. Additionally, IMiDs exert anti-angiogenic effects and disrupt stromal support within the bone marrow niche, further limiting MM cell growth.

In addition, IMiDs were found to contribute to an essential role in immune modulation, enhancing T-cell and natural killer (NK) cell activation while reducing regulatory T-cell function. Their integration into frontline and relapsed/refractory MM regimens, often in combination with proteasome inhibitors and monoclonal antibodies, has significantly improved overall survival. There is a large body of evidence that the gut microbiome can contribute to the immune system of cancer patients; thus, it can affect the IMiDs’ action [85]. Some microbiota species, such as *Lactobacillus* and *Akkermansia,* may produce a synergistic effect with IMiDs through the regulation of T cell differentiation and modulation of inflammation. On the other hand, dysbiosis could impair IMiDs’ action by preventing T cell function and enhancing inflammation. This could lead to shutting down the immune system’s ability to fight myeloma cells [86].

CAR-T cells and BiTEs are immunotherapies used in relapsed or refractory MM. CAR-T cells are engineered autologous T cells to target specific tumor antigens, such as B-cell maturation antigen (BCMA). BCMA-directed CAR-T cells recognize and eliminate MM cells through direct cytotoxicity, production of inflammatory cytokines, and induction of apoptosis. CAR-T cells have the ability to modify the T cell antigenic receptors, which in turn could target myeloma cells [87,88].

Moreover, BiTEs can bind the CD3 protein on T cells and tumor-associated antigens such as BCMA, GPRC5D, or FcRH5 on malignant plasma cells, enhancing the anti-cancer effect. As presented, CAR-T cells and BiTEs rely heavily on T cell function and activity. Therefore, the use of broad-spectrum antibiotics can lead to dysbiosis, compromising the immune cells of the patient and hindering the effectiveness of CAR-T cells and BiTEs [89].

### 5.1. Microbiome-Based Therapies in Multiple Myeloma

Recent scientific evidence supports a significant role for the gut microbiome in shaping immune responses and influencing cancer progression, including hematological malignancies like MM. Alterations in microbial diversity and composition (dysbiosis) have been associated with disease severity, treatment toxicity, and immune dysfunction in MM patients [90,91]. Consequently, microbiome-based therapies, including fecal microbiota transplantation (FMT), probiotics, and prebiotics, are emerging as novel adjuncts to conventional MM treatments, particularly in the era of precision medicine. The microbiome modulation therapies and their outcomes in MM management have been listed in Table 2.

#### 5.1.1. Fecal Microbiota Transplantation (FMT)

FMT involves the transfer of stool from a healthy donor into a recipient to restore a beneficial microbial composition. Initially established for the treatment of recurrent *Clostridioides difficile* infection, its application in oncology is growing due to its ability to reverse treatment-related dysbiosis and modulate anti-tumor immunity [92].

In MM, the gut microbiome is significantly disrupted following high-dose chemotherapy and autologous stem cell transplantation (ASCT) [20]. Dysbiosis in this context is linked with increased risk of bloodstream infections, mucosal barrier injury, and impaired immune reconstitution [91]. Preclinical and early clinical observations suggest that beneficial taxa such as *Eubacterium hallii* and *Faecalibacterium prausnitzii* enhance short-chain fatty acid (SCFA) production, promote immune regulation, and support T-cell and natural killer (NK) cell activation, thereby strengthening anti-myeloma immunity. Additionally, preclinical models suggest that FMT can restore microbial diversity and enhance CD^8+^ T cell function, leading to improved tumor control [93,94]. A study by Daillère et al. demonstrated that the efficacy of cyclophosphamide in MM-bearing mice was partially dependent on gut microbiota, particularly *Enterococcus hirae* and *Barnesiella intestinihominis*, which promoted immune-mediated tumor regression [95]. In another study, germ-free or antibiotic-treated mice showed inferior responses to immunotherapy unless they received FMT from responder patients [96]. These results support the immunomodulatory potential of FMT in MM.

**Table 2:** The microbiome modulation therapies and the associated outcomes in multiple myeloma (MM).

Therapy/Exposure Type	Type (Clinical/Preclinical)	Design	Sample Size (*n*)	Population/Model	Intervention/Exposure	Outcomes Measured	Key Findings	Adverse Events/Safety	Reference
Autologous FMT	Clinical	Prospective feasibility study	7	Adults undergoing HSCT	Re-infusion of the patient’s own pre-treatment stool (autologous FMT)	Feasibility, gut microbiota restoration, incidence of infections, GVHD, engraftment	Auto-FMT was feasible, safe, and restored gut microbiota diversity with early clinical benefits.	No FMT-related serious adverse events; overall well-tolerated	[97]
Fecal microbiota diversity changes after auto-HCT	Clinical	Prospective, multicenter observational cohort study	1325	Adults undergoing autologous HCT for hematologic malignancies (incl. multiple myeloma, lymphoma)	Fecal microbiome profiling (16S rRNA sequencing) before and after auto-HCT	Microbiota diversity (Shannon index), associations with overall survival, relapse, infectious complications	Gut microbiota diversity loss after auto-HCT predicted poorer survival and higher non-relapse mortality.	Not applicable (observational, sequencing only)	[98]
Gut microbiome perturbation and ASCT outcomes	Clinical	Prospective observational pilot study	30	Patients with multiple myeloma undergoing ASCT	Longitudinal fecal microbiome profiling (16S rRNA sequencing) during ASCT	Microbial diversity, engraftment, infectious complications, treatment response	Post-ASCT microbial diversity loss delayed engraftment, increased infections, and altered treatment response.	Not applicable (observational, sequencing only)	[41]
Gut microbiome alterations in MM	Clinical	Cross-sectional case–control study	37	Newly diagnosed MM patients vs. age-matched controls	Stool microbiome sequencing (16S rRNA & metagenomics)	Microbiome composition, metabolic pathways, and nitrogen metabolism	MM patients showed nitrogen-recycling bacteria enrichment, potentially accelerating disease progression.	Not applicable (observational, sequencing only)	[64]
Gut microbiome diversity and HSCT outcomes	Clinical	Prospective observational cohort study	80	Adults undergoing allogeneic HSCT (incl. MM)	Longitudinal fecal microbiome profiling (16S rRNA sequencing)	GVHD incidence, overall survival, and relapse rates	Higher gut microbial diversity post-HSCT predicted lower mortality and reduced GVHD incidence.	Not applicable (observational, sequencing only)	[99]
Microbiota diversity and allo-HSCT survival	Clinical	Multicenter observational cohort study (US, EU)	1362	Hematologic malignancies (incl. MM) undergoing allo-HSCT	Fecal microbiome profiling (16S rRNA sequencing)	Overall survival, GVHD, infections	Microbial diversity loss strongly predicted increased mortality post-HSCT.	Not applicable (observational, sequencing only)	[100]

Hematopoietic stem cell transplantation (HSCT), Fecal microbiota transplantation (FMT), Graft versus host disease (GVHD), Autologous stem cell transplantation (ASCT).

Clinical trials are now underway to assess FMT’s safety and efficacy in MM. For instance, the phase I trial NCT03772840 investigates FMT in patients undergoing ASCT and its impact on microbiome recovery and immune reconstitution [101]. Although promising, challenges such as donor standardization, risk of pathogen transmission, and long-term safety remain.

#### 5.1.2. Probiotics and Prebiotics

Probiotics and prebiotics offer non-invasive alternatives to FMT for modulating the gut microbiome. Probiotics are live microorganisms that confer health benefits, while prebiotics are non-digestible food components that selectively support the growth of beneficial microbes.

In MM, specific probiotic strains such as *Bifidobacterium longum* and *Collinsella* have shown promise in enhancing gut barrier function, reducing inflammation, and promoting anti-tumor immunity [20]. A. muciniphila, in particular, has been linked with enhanced responses to PD-1 blockade in solid tumors, potentially through stimulation of IL-12-dependent immune pathways [102].

Prebiotics like inulin and fructooligosaccharides (FOS) support SCFA-producing bacteria, which play critical roles in reducing inflammation and maintaining gut homeostasis [103,104]. SCFAs such as butyrate exert immunomodulatory effects by promoting regulatory Treg differentiation and enhancing mucosal integrity, features especially valuable in MM patients with compromised immunity [105].

**Figure 3:**
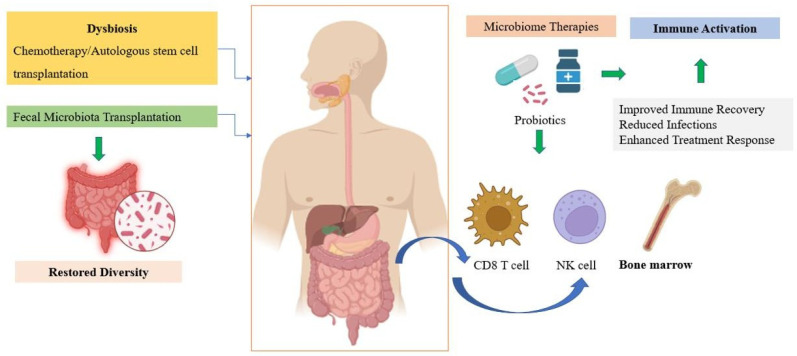
The microbiome-immune-myeloma cells interplay. Created in https://BioRender.com.

However, caution is necessary. Case reports of *Lactobacillus rhamnoses* bacteremia in immunocompromised patients have raised concerns about probiotic safety in MM [106]. Therefore, strain-specific safety evaluations are crucial before recommending probiotics in routine practice. Ongoing clinical trials assess the effects of microbiota-directed dietary interventions in MM, which may help identify safe, evidence-based recommendations for probiotic/prebiotic use (Figure 3).

#### 5.1.3. Personalized Medicine

Individual variations in microbiome composition influence treatment response, toxicity, and prognosis, making microbiome profiling a key component of personalized oncology. In MM, studies have shown that specific microbial taxa correlate with disease burden, progression, and immune competence. For example, a higher abundance of *Blautia* and *Faecalibacterium* was associated with favorable outcomes, whereas increased *Escherichia coli* and *Klebsiella pneumoniae* correlated with worse survival and elevated inflammatory markers [107].

Metagenomic sequencing enables the identification of patient-specific dysbiotic signatures, allowing for tailored microbial interventions. Personalized probiotics, selected based on an individual’s gut ecosystem, are under investigation for their ability to restore balance without causing harm. Similarly, individualized dietary regimens rich in specific prebiotics may help promote beneficial SCFA-producing bacteria.

Precision FMT, using defined bacterial consortia rather than whole fecal samples, is another emerging concept. This approach minimizes the risk of pathogen transmission and allows for standardized microbial therapy. For instance, defined microbial cocktails enriched in *Clostridiales* and *Lachnospiraceae* families could be engineered to modulate Tregs and anti-inflammatory cytokines [108,109].

In addition, microbiome-based biomarkers are being developed to predict treatment outcomes. For example, low alpha diversity and abundance of *Proteobacteria* were associated with increased risk of bloodstream infections post-ASCT in MM patients [110]. Early identification of such high-risk profiles could allow for proactive microbiome modulation.

By integrating microbiome data with host genomics, transcriptomics, and immune signatures, a systems biology approach can be developed for MM management. This precision medicine model holds the potential to optimize immunotherapy, minimize toxicity, and improve patient-specific outcomes. Microbiome-based therapies are transforming our approach to managing multiple myeloma. Through FMT, probiotics, prebiotics, and personalized modulation strategies, it is possible to recalibrate the gut-immune axis to support better immune recovery, reduce complications, and enhance therapeutic efficacy. Although challenges regarding safety, standardization, and regulatory approval persist, accumulating clinical and translational evidence strongly supports the integration of microbiome-targeted interventions into MM care.

### 5.2. Safety, Regulation, and Interactions of Microbiota with Standard MM Therapies

As previously discussed, gut microbiota has been found to affect MM disease progression, response to treatment (especially immunotherapies), and toxicity. Therefore, practical integration strategies such as using specific diets, prebiotics and probiotics, and FMT to modulate the gut ecosystem are encouraged. These strategies could significantly enhance efficiency and prevent treatment-related toxicities. Regarding safety, FMT induced some risk, including the possibility of infections, adverse reactions, and non-compliance; therefore, clinical trials and standardized procedures are required to address these issues.

Current microbiota-modulating interventions are generally considered safe in case of short-term treatment, but long-term safety data in MM patients remain scarce. Thus, regulatory protocols are rising, highlighting the safety and quality control standards of microbiota-based therapies to enable clinical adoption.

Moreover, inclusion of microbiota modulation into MM clinical management requires careful consideration of its interactions with standard MM therapies. The gut microbiota can affect the response and toxicity of standard MM treatments, including proteasome inhibitors (e.g., bortezomib, carfilzomib), immunomodulatory drugs (e.g., lenalidomide), corticosteroids (e.g., dexamethasone), monoclonal antibodies, and autologous hematopoietic stem cell transplantation (HSCT) [111]. Some SCFA–producing bacteria (i.e., *Faecalibacterium prausnitzii*) have been associated with decreased gastrointestinal toxicity and enhanced treatment efficacy; on the other hand, disruption of microbiota with the use could negatively affect the therapeutic responses of MM therapies [112].

The integration of microbiome modulation into clinical management of MM requires optimal timing of treatment cycles, ensuring safety, especially with MM patients’ immunocompromised status, and taking into consideration the patient’s microbiome variability.

## 6. Limitations of Current Studies

Current studies linking the role of the microbiome in MM disease progression and treatment showed some limitations. Many clinical studies on MM involved a relatively small number of MM patients. This could limit the statistical power of the study; therefore, studies with a larger number of patients are encouraged to validate observed associations and determine robust microbiota signatures relevant to MM treatment outcomes. Moreover, many studies depend on the mechanistic pathways suggested from murine/animal models of MM. These models provide valuable mechanistic insights, but there are differences in microbiota composition, immune system function, and tumor biology compared to humans. Another limitation is the lack of longitudinal data. Cross-sectional or retrospective clinical studies provide a single and limited time point regarding the microbiota. Longitudinal studies are required to study the microbiota dynamics throughout disease progression and treatment cycles of MM. In addition, MM patients frequently receive antibiotics and chemotherapy, both of which profoundly alter the gut microbiota. The microbiota composition changes in case of disease with or without treatment. Tracing these changes is important. Therefore, standardized data regarding the concomitant antibiotic/chemotherapy use is needed to reduce confounding influences.

## 7. Conclusions and Future Directions

There is a large body of evidence demonstrating the interplay between the gut microbiome and MM. The gut microbial ecosystem plays an essential role not only in immune regulation and inflammation but also in disease onset, progression, and treatment outcomes. Microbial dysbiosis and changes in the microbial-derived metabolites have been linked to critical changes in the tumor microenvironment and resistance to therapy in MM patients. Modulation of the gut microbiome through probiotics, prebiotics, or fecal microbiota transplantation represents a promising avenue that may improve MM treatment. However, despite ongoing preclinical and preliminary clinical findings, there is still a gap of knowledge in the link between the microbiome and MM. The exact mechanistic pathways linking the gut microbiome to MM onset and progression remain incompletely understood. There is an urgent need for established preclinical mechanistic studies and longitudinal clinical trials to better understand this interplay and develop personalized microbiome-based interventions. Longitudinal clinical trials with a larger number of patients are required to validate the findings. Also, the development of effective microbiota-based therapies requires a personalized approach due to individual microbiome variability. Furthermore, the multi-omics approach can be included in future studies to study microbiome composition differences and enhance clinical outcomes.

## Figures and Tables

**Table 1:** Microbial groups associated with multiple myeloma (MM).

Microbial Groups	Key Roles	Experimental Outcomes	References
Nitrogen-recycling bacteria (*Klebsiella pneumoniae*)	Produce glutamine, supplying the proliferating cells with their metabolic demand	Promoted cell growth and boosted the disease progression	[64]
*Prevotella heparinolytica*	Promotes intestinal Th17 cell differentiation and its migration to the bone marrow	Increased IL-6 releases Activated eosinophils, induced STAT3 phosphorylation and reduced IL-17 synthesis in bone marrow causes inflammation-mediated MM progression	[39]
SCFA-producing bacteria such as (*Eubacterium halii*, *Faecalibacterium prausnitzii*, and *Clostridium butyricum*)	Produce SCFA as butyrate, propionate, and acetate that maintain the intestinal barrier integrity and hinder NF-κB activation and inflammation	Their depletion in MM resulted in: Decreased SCFA levels Reduced tight junctions and loss of intestinal barrier integrity Increased inflammation	[65,66]

## Data Availability

No new data were created or analyzed in this study.
